# In vitro and in vivo antibacterial effect of NZ2114 against *Streptococcus suis* type 2 infection in mice peritonitis models

**DOI:** 10.1186/s13568-017-0347-8

**Published:** 2017-02-20

**Authors:** Jian Jiao, Ruoyu Mao, Da Teng, Xiumin Wang, Ya Hao, Na Yang, Xiao Wang, Xingjun Feng, Jianhua Wang

**Affiliations:** 1 Key Laboratory of Feed Biotechnology, Ministry of Agriculture, Beijing, 100081 People’s Republic of China; 2 Gene Engineering Laboratory, Feed Research Institute, Chinese Academy of Agricultural Sciences, 12 Zhongguancun Nandajie Street, Haidian District, Beijing, 100081 People’s Republic of China; 3 Institute of Animal Nutrition, Northeast Agricultural University, Harbin, 150030 People’s Republic of China

**Keywords:** Antimicrobial peptides, NZ2114, *Streptococcus suis*, Anti-*S. suis* in vivo

## Abstract

NZ2114 is a promising candidate for therapeutic application owing to potent activity to gram-positive bacterium such as *Streptococcus pneumoniae* and *Staphylococcus aureus*. This work is the first report to describe the in vitro and in vivo antibacterial characteristics of NZ2114 against *Streptococcus suis*. It exhibited strong antimicrobial activity against *S. suis* type 2 strains CVCC 606, CVCC 3309, and CVCC 3928 at a low minimal inhibitory concentration (MIC) of 0.03–0.06 μM. The NZ2114 killed over 99.9% of tested *S. suis* CVCC 606 in Mueller–Hinton medium within 4 h when treated with 4 × MIC. It caused only less than 0.25% hemolytic activity in the concentration of 256 μg/ml. Additionally, NZ2114 exhibited potent in vivo activity to *S. suis*. All mice were survival when the dosage was low to 0.2 mg/kg. Over 99% of *S. suis* cells were killed within 4 h in blood, lung, liver and spleen with dosage of 10, 20, and 40 mg/kg in mice peritonitis models and no pathogen were detected after 24 h of treatment. Further, no pathological phenomenon in lung and low level of inflammatory cytokines in blood were detected. These results indicate that NZ2114 has the potential to be a new antimicrobial agent candidate for the clinical treatment of infection caused by *S. suis* type 2.

## Introduction

**Fig. 1:**
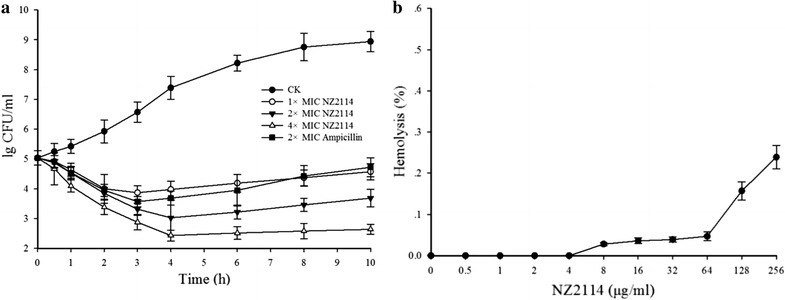
Time-killing curves and hemolytic assay of NZ2114. **a** Time-killing curves of NZ2114 against *S. suis* CVCC606. *S. suis* CVCC606 were incubated in the presence of medium alone (CK), in the presence of NZ2114 at 1×, 2×, and 4× MIC, or in the presence of ampicillin at 2 × MIC, three duplicate observations were made; *bars* represent the standard error of the mean. **b** Hemolytic assay of NZ2114. The 4% erythrocytes were incubated with different concentration of NZ2114, the 0.9% NaCl and 0.1% Triton X-100 were used as negative and positive controls. Three duplicate observations were made; *bars* represent the standard error of the mean

*Streptococcus suis* is an important pathogen associated with wide range of diseases in swine and human, including septicemia, pneumonia, endocarditis, meningitis, and arthritis (Lun et al. 2007). It was found that *S. suis* was the fourth most significant pathogen in the breeder and weaner site. *S. suis* can be transmitted to human beings by direct contact. The repeated intensive outbreaks of human *S. suis* infection have raised great public concern worldwide regarding *S. suis* as an emerging zoonotic pathogen. It is the most common cause of adult infection in Vietnam (Mai et al. 2008) and the second most common in Thailand (Suankratay et al. 2004). In Europe, the largest number of zoonotic infections due to *S*. *suis*, have been recorded in the Netherlands [Figure 1 in (Wertheim et al. 2009)]. In July, 2005, the largest outbreak of human *S. suis* infection occurred in Sichuan province, China, where 204 people were infected and 38 of them died (Normile 2005). There are 35 serotypes described and the composition of the capsule defines the serotype (types 1–34 and 1/2). Serotype 2 is commonly associated with diseases in pigs and human beings, and is the most frequently reported serotype worldwide (Costa et al. 2005). It has always been considered the most virulent serotype (Higgins and Gottschalk 2000).

*S. suis* type 2 is resistant to various environmental conditions. It can survive for 10 min at 60 °C, 2 h at 50 °C, and 6 weeks in carcasses at 10 °C (Clifton-Hadley and Enright 1984). The penicillin G, accompanied by one or more other antibiotics including ceftriaxone, gentamicin, chloramphenicol, and ampicillin is the normal strategy for *S. suis* infection treatment (Halaby et al. 2000). However, with wide and over use of antibiotics, *S. suis* is resistant to many conventional drugs. More than 87% of *S. suis* isolates are  resistant to oxytetracycline, erythromycin, tylosin tartrate, and clindamycin in Spain (Vela et al. 2005). In addition, high levels of tetracycline resistance (upto 90%) have also been reported from diseased and clinically healthy persons (Hoa et al. 2011; Strangmann et al. 2002). Integrative conjugative elements (ICE) seem to play a key role in the transmission of resistance determinants, as demonstrated by genomic studies. Although *S. suis* is uniformly sensitive to penicillin or ampicillin, and low levels of resistance are reported (Varela et al. 2013), there will be rare candidates when resistance arise. In the other side, vaccines are the common strategy for the prevention of *S. suis* infection. Although various of vaccines are developed, the commonly used vaccines in pig industry, however, remain the inactivated autogenous vaccine generated from virulent strains isolated from sick pigs (Haesebrouck et al. 2004). One of the disadvantages of autogenous vaccines is the absence of safety and efficacy data. At present, there is no *S. suis* vaccine for human beings (Lun et al. 2007). To prevent and treat infections caused by *S. suis*, novel and effective antimicrobial agents are needed.

Plectasin is a fungal defensin from *Pseudoplectania nigrella* and is active against Gram-positive bacteria such as *Staphylococcus aureus* (MIC_50_ 16 μg/ml for methicillin-sensitive strains and 32 μg/ml for resistant strains) and *S. pneumoniae* (MIC_50_ 1 μl/ml for both penicillin-sensitive and resistant strains) by coalescing with the pyrophosphate moiety of lipid II, the essential precursor of the cell wall (Mygind et al. 2005; Schneider et al. 2010). Peptide NZ2114 is a novel variant of plectasin (D9N, M13L, Q14R) that is significantly more potent than parental peptide (MIC_50_ 2 μg/ml for *S. aureus* and 0.25 μg/ml for *S. pneumoniae*) (Andes et al. 2009; Ostergaard et al. 2009; Zhang et al. 2014). It also owned long postantibiotic effect (PAE) (Andes et al. 2009) and was synergistic in combination with teicoplanin, moenomycin, and dalbavancin (Breidenstein et al. 2015). It had potent activities against *S. aureus* in rabbit meningitis, murine peritonitis, and thigh infection models (Andes et al. 2009; Ostergaard et al. 2009; Xiong et al. 2011). Additionally, NZ2114 showed low or no cell toxicities, long-lasting serum stability and in vivo half-life (Brinch et al. 2010). However, there are no studies focused on its activity against *S. suis*, the important zoonotic pathogens. In this work, the in vitro effect of NZ2114 against *S. suis* was investigated. Furthermore, the in vivo pharmacodynamics characteristics were evaluated for future clinical development.

## Materials and methods

### Materials

The antimicrobial peptide NZ2114 was prepared according to a previously described protocol (Zhang et al. 2014), and its purity was 94.8%. The purified, lyophilized NZ2114 powder was dissolved in sterilized ultrapure water and stored at −20 °C before subsequent antibacterial assessments.

### Minimal inhibitory concentration (MIC) assay

The MICs of NZ2114 were determined by the microtiter broth dilution method in 96 micro-well plate (Tian et al. 2009). The pathogens were grown to 0.4 of OD_600nm_ at 37 °C in MHB medium and diluted to approximate 1 × 10^5^ CFU/ml with fresh MHB medium. The purified NZ2114 was twofold serial dilutions with gradient concentration of 1280, 640, 320, 160, 80, 40, 20, 10, 5, 2.5, 1.25, and 0.625 μg/ml. A 90-μl cell suspension and 10 μl of serial concentration gradient solutions of NZ2114 were added to every well. All assays were performed in triplicate. The ampicillin was also tested with the same concentration gradient as controls. Plates were incubated at 37 °C for 18–24 h. The MIC was defined as the lowest concentration of ones at which there was no visible growth.

### Bactericidal kinetics assay

The *S. suis* strain CVCC 606 was grown overnight in MH medium at 37 °C with shaking at 250 rpm. Fresh MH medium was inoculated with 1% (v/v) overnight culture and grown to mid-log phase. The 90 μl of exponential-phase *S. suis* strain CVCC 606 (approximately 10^4−5^ CFU/ml) cells were incubated with 10 μl of NZ2114 (final concentrations were 1×, 2×, and 4 × MIC); the 2 × MIC ampicillin was used as control. The mixed samples were added to the wells of 96-well cell culture plates (each concentration sample was performed in triplicate) and incubated at 37 °C. The 100 μl samples from each well were collected after 0, 0.5, 1, 2, 3, 4, 6, 8, and 10 h of incubation and were serially diluted and plated on MH agar. Viable colonies were counted after 16–18 h at 37 °C.

### Hemolytic assay

The hemolytic activity of NZ2114 was evaluated by determining the amount of released hemoglobin from a 4% suspension of fresh mice red blood cells (RBCs) (Cho and Lee 2011). Mice RBCs were collected and washed with physiological saline (PS) three times. The 100 μl of mice RBCs diluted to 8% (v/v) in PS was seeded into 96-well plates, and a 100 μl peptide solution was then added to each well (at a final concentration ranging from 0 to 256 μg/ml). The plates were incubated at 37 °C for 1 h and centrifuged at 1500 rpm for 10 min. Absorbance of the supernatants at 540 nm was measured with an ELISA plate reader, and 0 and 100% hemolysis was determined in PS and 0.1% Triton X-100, respectively. The hemolysis percentages were calculated by the following equation: [(Abs_540nm_ in NZ2114 solution − Abs_540nm_ in PS)/(Abs_540nm_ in 0.1% Triton X-100 − Abs_540nm_ in PS)] × 100%.

### Animals

The 6-week-old female Institute for Cancer Research (ICR) mice, SPF, weighing 20–25 g were used for all in vivo test. Mice were purchased from Wei Tonglihua Co., Ltd (Beijing). Animals were kept in standard Macrolon cages (5–8 per cage), fed a standard pellet diet ad libitum, and had free access to bottled drinking water. Animals were acclimatized for 2–3 days prior to the initiation of the study.

### *Streptococcus suis* infection model

*Streptococcus suis* CVCC606 bacteria were grown to logarithmic phase (OD_600nm_, 0.5), harvested, washed in PBS, diluted in the same buffer to 8.5–9.5 lg CFU/ml, and kept on ice until injection. Every ten mice were divided into a group. A 100 μl of certain concentration of bacterial suspension was inoculated intraperitoneally (i.p.). The PBS was used as control. After injection of bacteria, survival rate was monitored for 7 days.

### Effects of NZ2114 against *S. suis* in vivo

To test the protective effect of NZ2114 against *S. suis* infections in vivo, every ten ICR female mice were divided into one group. Each group was inoculated i.p. with 1 × 10^8^ CFU *S. suis* CVCC606 and various concentration of NZ2114 (0.04, 0.2, 1, 2.5, 5, 10, 20, and 40 mg/kg) were injected intravenously (i.v.) after 1 and 8 h post-infection. The PBS and 10 mg/kg ampicillin was used as control. After injection of bacteria and drugs, survival rate was monitored for 7 days. Additionally, the blood and lung were collected after 24 h post-treatment; bacterial loads in these tissues were calculated.

The bacterial loads in different tissues after treatment with NZ2114 at different time points were determined. ICR female mice inoculated with 1 × 10^8^ CFU *S. suis* CVCC606 and various concentration (10, 20, and 40 mg/kg) of NZ2114 was injected i.v. after 1 and 8 h post-infection. The mice were sacrificed by cervical dislocation; bacterial loads in blood, lung, spleen, and liver were detected in 0, 2, 4, 8, and 16 h post-treatment. Every six mice were divided into one group and as duplicate.

### Histological analysis of lung

ICR female mice were inoculated with 1 × 10^8^ CFU *S. suis* CVCC606 and various concentration of NZ2114 (10, 20, and 40 mg/kg) was injected after 1 and 8 h post-infection. The 10 mg/kg ampicillin was used as control. The mice were sacrificed after 24 h post-treatment and the lungs were removed and immediately dipped into 4% paraformaldehyde solution in PBS for 1–3 days. The tissues were then embedded in paraffin, sectioned and 4 mm sections were placed on glass slides. Slides underwent deparaffinization and staining by hematoxylin and eosin.

### Cytokine assay

ICR female mice were inoculated with 1 × 10^8^ CFU *S. suis* CVCC606 and various concentration of NZ2114 (10, 20, and 40 mg/kg) was injected after 1 and 8 h post-infection. The 20 mg/kg ampicillin was used as control. Whole blood was collected by heart puncture after 24 h post-treatment. The blood was incubated at 37 °C for 2 h and centrifuged at 3000 rpm for 5 min at 4 °C. Serum was collected from supernatant. The cytokines of TNF-α, IL-6 and IL-10 was detected by ELISA in Jiaxuan Biotech Co., Ltd (Beijing).

## Results

### Antimicrobial activity of NZ2114

The NZ2114 displayed potent antimicrobial activity against Gram-positive bacteria such as *S. suis*, *S. aureus* and *S. pneumonia*, especially for *S. suis* (MIC 0.03–0.06 μM) (Table 1). The MBCs ranged from 0.06 to 0.12 μM. The activity of NZ2114 to *S. aureus* and *S. pneumonia* was lower than that to *S. suis*, with the MICs ranged from 0.03 to 0.9 μM to *S. aureus* ATCC25923, 6538 and 43300 and 1.8–3.6 μM to *S. pneumonia* CMCC31968 and 2350, respectively (Table 1). In addition, the activity of NZ2114 against *S. suis* was stronger compared to ampicillin (MIC 0.17–0.34 μM) (Table 1).

**Table 1:** MIC and MBC assays of NZ2114 and ampicillin to *S. suis*, *S. aureus*, *S. pneumonia*, and *E. coil*

Strains	Source	MIC (μM)		MBC (μM)	
		NZ2114	Ampicillin	NZ2114	Ampicillin
*S. suis* CVCC606	CVCC^a^	0.03	0.17	0.03	0.34
*S. suis* CVCC3309	CVCC	0.03	0.34	0.06	0.34
*S. suis* CVCC3928	CVCC	0.06	0.34	0.06	1.02
*S. aureus* ATCC25923	CVCC	0.03	1.35	0.06	1.35
*S. aureus* ATCC6538	CGMCC^b^	0.11	1.35	0.11	2.69
*S. aureus* ATCC43300	CGMCC	0.9	10.78	0.9	21.56
*Streptococcus pneumoniae* CGMCC1.8722	CGMCC	0.45	1.35	0.9	2.69
*S. pneumoniae* CVCC2350	CVCC	0.9	2.69	1.8	5.38
*S. pneumoniae* CVCC31968	CVCC	0.9	2.69	0.9	2.69
*E.coli* CVCC195	CVCC	≥29.09	0.31	≥29.09	0.31
*E*.*coli* CVCC1515	CVCC	≥29.09	NT	≥29.09	NT

*NT* no tested

^a^China Institute of Veterinary Drug Control

^b^China General Microbiological Culture Collection Center

### Time-killing curves of NZ2114

Time-killing curves were generated to demonstrate the bactericidal ability of NZ2114 against *S. suis* CVCC606. As shown in Fig. 1a, in the absence of NZ2114, the bacterial counts (lg CFU/ml) reached to 8.92 at 10 h. It exerted a dose-related pattern of inhibition for *S. suis* CVCC606. A decrease in *S. suis* CVCC606 of 1.28 and 1.83 lg CFU/ml (>90% reduction) was observed within 3 h at one and two times of MIC, respectively. Those decreases were nearly equal to that obtained by ampicillin treatment (1.49 lg CFU/ml decrease) at two times of MIC (Fig. 1a), but NZ2114 failed to inhibit bacterial regrowth after 3 and 4 h of inoculate for 1× and 2 × MIC, respectively. However, a huge and stable decrease was found within 10 h at 4 × MIC (lg CFU/ml from 5.13 to 2.64, bactericidal efficiency >99%).

### Hemolytic assay

The cytotoxicity of NZ2114 was tested by measuring its ability to lyse mice RBCs. There was no obvious hemolysis of RBCs within 64 μg/ml and the hemolytic activities at concentrations of 128 and 256 μg/ml were 0.153, 0.241%, respectively (Fig. 1b). It is critical that NZ2114 displays little or no hemolytic activity for its application in internal medicine.

### Absolute lethal dose assay

**Fig. 2:**
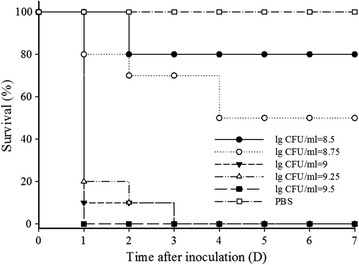
Absolute lethal dose assays. Every ten mice were divided into a group. Each group was injected with a certain concentration of *S. suis* CVCC606 (100 μl of 8.5–9.5 lg CFU/ml). The PBS was used as control. After injection of bacteria, survival rate was monitored for 7 days

As shown in Fig. 2, all mice were alive when i.p. injected with PBS after 7 days. The survival rate of mice injected with 8.5 and 8.75 lg CFU/ml *S. suis* CVCC606 were 80 and 60%, respectively. All mice were died at 7 days after injected with 9, 9.25, and 9.5 lg CFU/ml *S. suis* CVCC606. However, the mice injected with 9.5 lg CFU/ml bacteria were died immediately within only 1 day, and the mice injected with 9 and 9.25 lg CFU/ml bacteria were died at 3 days postinjection. To evaluate the therapeutic effects in a continuous way, the inoculum concentration of 9 lg CFU/ml was chosen to further research.

### Dosage effect of NZ2114

**Fig. 3:**
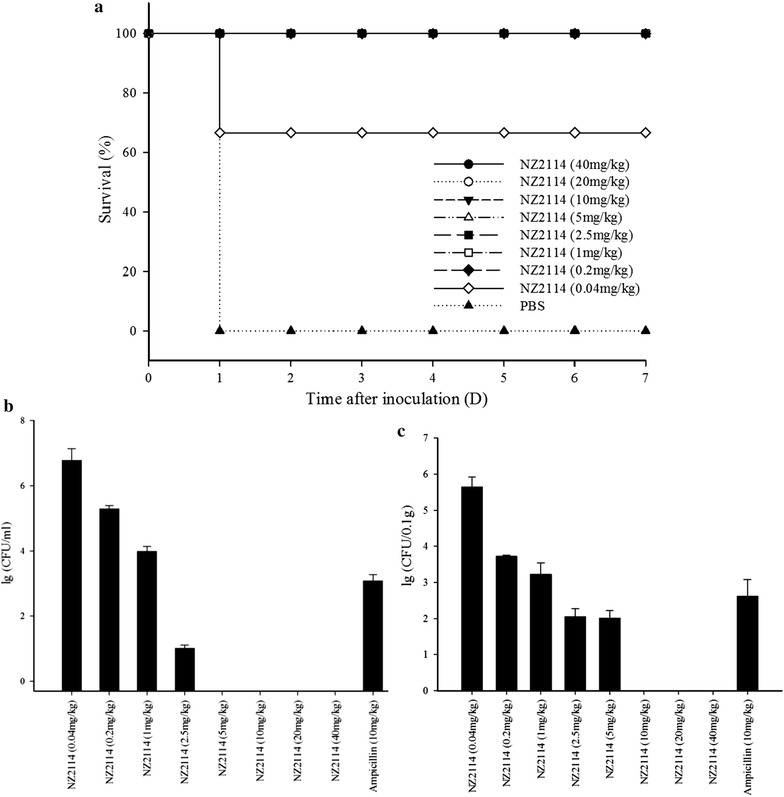
Dosage effect of NZ2114. **a** Effects of NZ2114 on the survival rate of mice. Every ten ICR female mice were divided into one group. Each group was inoculated with 1 × 10^8^ CFU *S. suis* CVCC606 and various concentration of NZ2114 was injected after 1 and 8 h post-infection. The PBS was used as control. After injection of bacteria, survival rate was monitored for 7 days; **b** Effects of NZ2114 on the bacterial loads in blood after 24 h post-treatment. Every six mice were divided into a group and as duplicate, *bars* represent the standard error of the mean; **c** Effects of NZ2114 on the bacterial loads in lung after 24 h post-treatment. Every six mice were divided into a group and as duplicate, *bars* represent the standard error of the mean

It was shown that NZ2114 improved survival for all treatment groups (Fig. 3a). All mice injected with 0.2–40 mg/kg NZ2114 were alive except that of 0.04 mg/kg (Fig. 3a). No obvious colony was observed in the blood of mice injected with 5–40 mg/kg NZ2114. However, the lg CFU/ml of *S. suis* CVCC606 increased significantly from 0.74 to 6.50 with the NZ2114 concentration of 2.5, 1, 0.2 and 0.04 mg/kg, respectively (Fig. 3b). In addition, NZ2114 with the concentration of 10, 20, 40 mg/kg could effectively kill the pathogens and no colony was observed in lung after 24 h of injection. The lg CFU/ml of *S. suis* CVCC606 was 2.01 and 2.04 with the dosage of 5 and 2.5 mg/kg and it increased from 3.23 to 5.64 with the reduction of injection dose (1, 0.2, and 0.04 mg/kg) (Fig. 3c).

### Effects of NZ2114 against *S. suis* on various organs

**Fig. 4:**
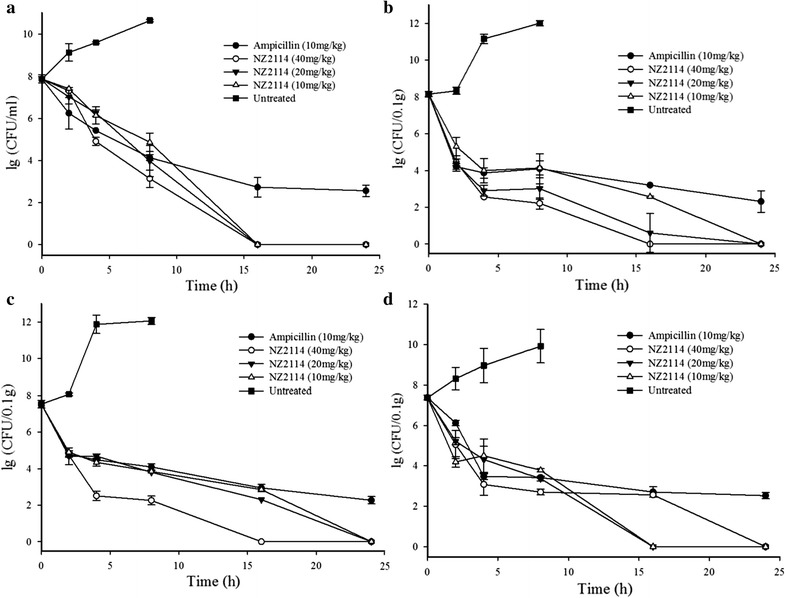
Effects of NZ2114 on bacterial loads of *S. suis* in various organs. ICR female mice was inoculated with 1 × 10^8^ CFU *S. suis* CVCC606 and various concentration (10, 20, and 40 mg/kg) of NZ2114 was injected after 1 and 8 h post-infection. The bacterial loads in blood (**a**), lung (**b**), spleen (**c**), and liver (**d**) were detected in 0, 2, 4, 8, and 16 h post-treatment. Every six mice were divided into one group and as duplicate, *bars* represent the standard error of the mean

The bacterial loads in different organs were monitored at 2, 4, 8, 16, and 24 h postinfection after treatment with NZ2114 and control ampicillin. *S. suis* was distributed at high concentrations in different organs and blood at 4 h (8.97–11.87 lg CFU/ml) and 8 h (9.92–12.06 lg CFU/ml) postinfection. Meanwhile, the bacterial loads in blood showed sharp decrease and hardly detected at 16 h posttreatment with 10, 20, and 40 mg/kg NZ2114. The ampicillins with 10 mg/kg led to 5.313 lg CFU/ml reduction in 24 h postinfection (Fig. 4a).

The bacterial loads in lung, spleen, and liver showed obvious dose-dependent effect (Fig. 4b–d). There was significant reduction of the CFU counts in lung, with the most rapid decrease being observed in 4 h posttreatment, the bacterial loads were 2.560, 2.903, and 3.995 lg CFU/0.1 g with 40, 20 and 10 mg/kg NZ2114, respectively. However, the bacterial loads remained stable until the retreatment in 8 h and no colony were detected in 24 h posttreatment (Fig. 4b). Similarly, there was significant reduction of the CFU counts in 4 h posttreatment, showing lg colony counts of 2.512, 4.345, 4.689 in spleen and 3.078, 4.313, 4.506 in liver for 40, 20, and 10 mg/kg NZ2114, respectively (Fig. 4c, d). Moreover, there was no colony in 24 h at all NZ2114 treatment groups.

### Effects of NZ2114 against *S. suis* infection in lung histopathology

**Fig. 5:**
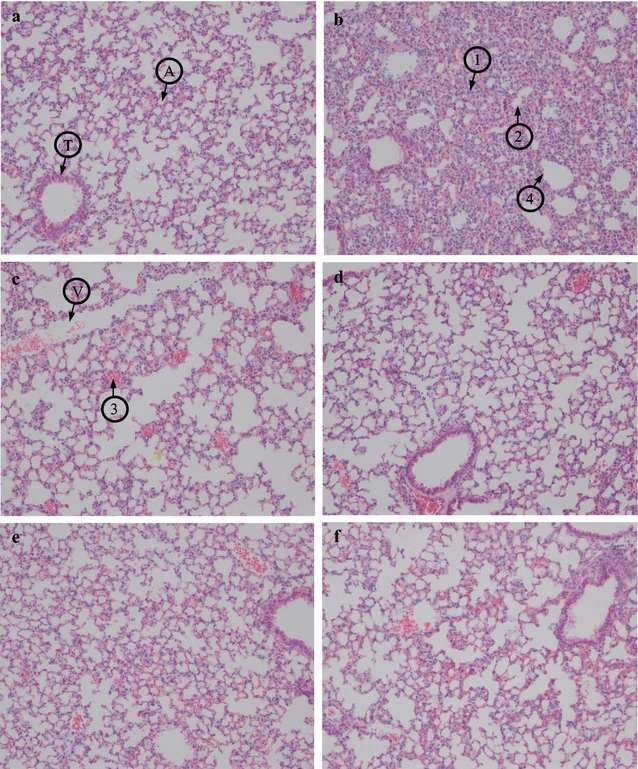
Lung histopathology from different NZ2114 concentration experimental groups. ICR female mice were inoculated with 1 × 10^8^ CFU *S. suis* CVCC606 and various concentration of NZ2114 (10, 20, and 40 mg/kg) was injected after 1 and 8 h post-infection. The mice were sacrificed after 24 h post-treatment and lung tissue was collected for histological evaluation. **a** Control group; **b** *S. suis* CVCC606 infection group; **c** *S. suis* CVCC606 + 10 mg/kg NZ2114; **d** *S. suis* CVCC606 + 20 mg/kg NZ2114; **e** *S. suis* CVCC606 + 40 mg/kg NZ2114; **f** *S. suis* CVCC606 + 20 mg/kg ampicillin. *A* alveoli, *T* bronchioli terminals, *V* vessel, *1* infiltration of inflammatory cells, *2* alveolar collapse, *3* alveolar hemorrhage, *4* bronchioli terminals epithelium damage

There was no histological sign of infection and inflammation in the lung tissue of uninfected group (Fig. 5a). In contrast, the histology of untreated mice showed severe inflammatory reactions such as infiltration of inflammatory cells, alveolar collapse, alveolar hemorrhage and bronchioli terminals epithelium damage (Fig. 5b). NZ2114 could reduce the lesions of lung tissues in a dose dependent manner (Fig. 5c–e). There was no obvious inflammation in the 20 and 40 mg/kg NZ2114 treatment group, which was recovered almost the same as uninfected group (Fig. 5d, e).

### Effects of NZ2114 in the cytokine levels

**Fig. 6:**
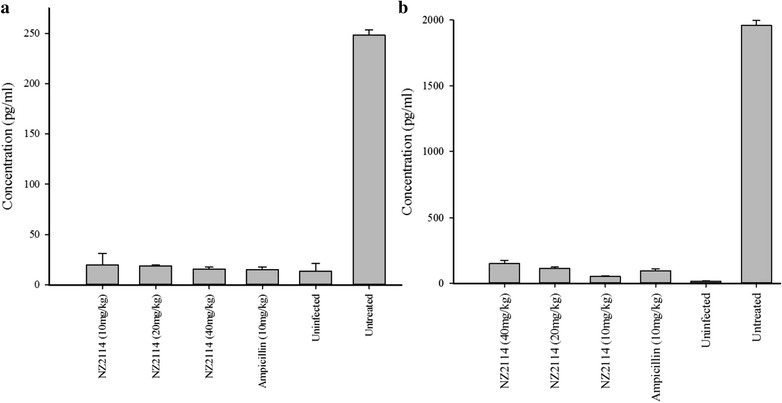
Effects of NZ2114 on the pro-inflammatory cytokines of TNF-α and IL-6 in serum. ICR female mice were inoculated with 1 × 10^8^ CFU *S. suis* CVCC606 and various concentration of NZ2114 (10, 20, and 40 mg/kg) was injected after 1 h post-infection. The mice were sacrificed after 24 h post-treatment and serum was collected for cytokines of TNF-α (**a**) and IL-6 (**b**) analysis. Three duplicate observations were made; *bars* represent the standard error of the mean

To expose the anti-inflammatory effect of NZ2114 on mice, the levels of inflammatory cytokines TNF-α, IL-6 and IL-10 were analyzed after 24 h post-treatment. As shown in Fig. 6, there were low cytokine levels in the uninfected group (13.798 pg/ml for TNF-α and 16.256 pg/ml for IL-6). The *S. suis* infection could significantly increase cytokine levels to 248.19 and 1458.60 pg/ml, respectively (Fig. 6a, b). Conversely, treatment with NZ2114 efficiently reduced the production of TNF-α and IL-6, reducing to the normal levels (15.679–19.671 pg/ml for TNF-α and 52.666–149.813 pg/ml for IL-6) (Fig. 6a, b). In contrast, no obvious changes of expression of IL-10 were exhibited in control and treatment groups (data not shown).

## Discussion

Antimicrobial peptides (AMPs) have attracted much attention in recent years for their potent activity against a variety of pathogens, including drug-resistant bacteria. Currently, 2718 types of AMPs are registered in the antimicrobial peptide database (APD) (http://aps.unmc.edu/AP/main.php). However, only a few of them advanced into clinical trials due to several bottlenecks, including poor antimicrobial activity, low stability, high toxicity and lack of efficient approaches to commercial-scale production (Eckert 2011; Yeung et al. 2011; Zasloff 2002). NZ2114 was a novel plectasin mutant identified through a high-throughput mutation, which had improved potency against *S. aureus* and *S. pneumoniae* (Andes et al. 2009; Ostergaard et al. 2009) and high expression yield (2.39 g/l in the supernatant of *Pichia pastoris*) (Zhang et al. 2014). Our previous studies showed that, plectasin and its derived peptide MP1106 were very active against G^+^ pathogens, such as *S. suis*, *S. aureus* and *S. pneumonia*, especially for *S. suis* (MIC 0.03–0.06 μM) (Cao et al. 2014; Jiao et al. 2015; Zhang et al. 2011). However, there was no detailed research on the in vitro and vivo characteristic of NZ2114 against *S. suis*—the pathogen of zoonosis got great public attentions in recent years. In this work, NZ2114 had the best activity against *S. suis* (MIC 0.03–0.06 μM) (Table 1). In addition, it could kill 99% pathogens within 4 h at 2 and 4 × MICs (0.06–0.12 μM), having no bacterial regrowth after 4 h of inoculate (Fig. 1a). The MP1106 also significantly inhibited the growth of pathogen but was in high concentration (0.24 and 0.48 μM for 4 and 8 × MICs), there was no obvious bacterial regrowth only in the 8 and 16 × MICs (Jiao et al. 2015). The more excellent anti-*S. suis* characteristic makes it as a potential antimicrobial drug for *S. suis* infection treatment.

The hemolysis was often evaluated for candidate AMPs as a new agent through intravenous injection (Park et al. 2006; Tian et al. 2009). NZ2114 and plectasin have been proven to have no hemolysis to human erythrocytes (less than 0.1% in 128 μg/ml) and no toxicity to A549 cells, normal human bronchial epithelial cells, or lung fibroblasts in 50 μg/ml (Hara et al. 2008; Mygind et al. 2005; Zhang et al. 2014). As expected, NZ2114 also showed a very low hemolytic activity (<0.3%) against mouse erythrocytes even in the high concentration of 256 μg/ml (Fig. 1b). The low toxicity to both model animal and human erythrocytes make it convenient for intravenous injection for further research and clinical use.

The plectasin and NZ2114 had potent activity in different animal infection models. The in vivo efficacy of plectasin (10 mg/kg) was comparable to vancomycin (70 mg/kg) in the peritoneal model and to penicillin (30 mg/kg) in pneumonia model caused by *S. pneumoniae* (Mygind et al. 2005). It was shown that NZ2114 (10, 40 and 160 mg/kg) could effectively killed *S. aureus* and *S. pneumoniae* in neutropenic murine thigh infection model. The counts of pathogen decreased over 99% within 5 h. There was regrowth with 10 mg/kg NZ2114 in *S. pneumoniae* group and all dosage in *S. aureus* group after 5 h of therapy (Andes et al. 2009). Additionally, there was a significantly higher level of cerebrospinal fluid (CSF) penetration of NZ2114 through inflamed than through non inflamed meninges. Treatment with NZ2114 (40 and 20 mg/kg), caused a significantly higher reduction in CSF bacterial concentrations than therapy with ceftriaxone (125 mg/kg) at 3, 5 and 10 h (Ostergaard et al. 2009). In this work, NZ2114 showed strong activity to *S. suis*. The 0.2 mg/kg of it could save all infected mice (Fig. 3a) and there was no pathogen in blood (5 mg/kg) and lung (10 mg/kg) (Fig. 3b, c). *S. suis* loads of organs further revealed that there were no pathogen existed in blood, lung, liver and spleen after 24 h of treatment (twice injection, 1 and 8 h of postinfection) in all dosages (10, 20 and 40 mg/kg) (Fig. 4). Other antimicrobial agents had the same effects but in high dosage. The 80 mg/kg (calculated at 25 g per rat) Ply30 (the bacteriophage lysin) protected 80% (8/10 mice) of mice from infection with *S. suis*. Seven days after lysin Ply30 treatment, bacterial loads were significantly decreased in all tested organs and blood compared with control group (Tang et al. 2015). It was shown that 8.6 and 28.02 mg/kg of cefquinome was required for 1-lg killing of *S. suis* ATCC 43765 with the initial inoculum of 10^8^ and 10^6^ CFU, respectively (Guo et al. 2016). The low injection dosage and high efficiency of NZ2114 in the mice model indicates that the drug resistance will be rare and it is suitable for the clinical treatment of *S. suis* infection.

Pulmonary injury could be the direct cause of *S. suis* -induced animal death (Lun et al. 2007). Lung inflammation is characterized by increased pulmonary inflammatory cell sequestration, which leads to the development of protein leakage in alveolar space, reduced lung compliance, and finally impaired lung function (Chu et al. 2010). It was shown that NZ2114 could reduce the lesions of lung tissues such as alveolar collapse and alveolar hemorrhage in a dose dependent manner, indicating that it is also conducive to the recovery of pulmonary injury.

TNF-α is the first endogenous cytokine released from monocytes and macrophages, which induces the inflammatory cascade and aggravates injuries (Malleo et al. 2007). Additionally, it leads to apoptosis by activating the caspases enzyme system in endothelial cells which compromises the integrity of the vascular barrier (Goldblum and Sun 1990). IL-6 is also a vital cytokine with strong influences on inflammatory responses and a crucial indicator (Strandberg et al. 2005). IL-10 has emerged as a key immunoregulator during infection. It can directly regulate innate and adaptive Th1 and Th2 responses by limiting T cell activation and differentiation in the lymph nodes as well as suppressing proinflammatory responses in tissues, leading to impaired pathogen control and/or reduced immunopathology (Couper et al. 2008). In this work, NZ2114 clearly reduced the levels of TNF-α, and IL-6 compared to the infection group (Fig. 6). These findings suggest that the protective effect of NZ2114 is partly attributed to inhibit the levels of pro-inflammatory cytokines. However, the level of anti-inflammatory IL-10 was ruleless (data not shown). Considering that IL-10 is pleiotropic functions, further studies are necessary to evaluate the immune state.

In conclusion, NZ2114 showed strong antimicrobial activity against Gram-positive bacteria, especially to *S. suis*. It had very low hemolysis to mice RBCs in safe application concentrations. It was also found that NZ2114 had potent in vivo activity to *S. suis*. All mice were survival when the dosage was low to 0.2 mg/kg and no pathogens were detected in blood, lung, liver and spleen with dosage of 10, 20 and 40 mg/kg after 24 h of treatment. Further, no pathological phenomenon in lung and low level of inflammatory cytokines in blood were detected. Above all, the in vitro and in vivo bactericidal profiles of NZ2114 indicating it is an ideal candidate for a new antimicrobial agent against *S. suis* infection.

## Abbreviations

MIC: minimal inhibitory concentration
RBCs: red blood cells
PS: physiological saline
ICR: institute for cancer research
i.p.: intraperitoneally
i.v.: intravenously
AMP: anitimicrobial peptide
APD: antimicrobial peptide database
CSF: cerebrospinal fluid
